# Comparing late‐onset epileptic spasm outcomes after corpus callosotomy and subsequent disconnection surgery between post‐encephalitis/encephalopathy and non‐encephalitis/encephalopathy

**DOI:** 10.1002/epi4.12698

**Published:** 2023-02-06

**Authors:** Takeshi Inoue, Ichiro Kuki, Takehiro Uda, Noritsugu Kunihiro, Ryoko Umaba, Saya Koh, Megumi Nukui, Shin Okazaki, Hiroshi Otsubo

**Affiliations:** ^1^ Department of Pediatric Neurology Osaka City General Hospital Osaka Japan; ^2^ Department of Pediatric Neurosurgery Osaka City General Hospital Osaka Japan; ^3^ Department of Neurosurgery Osaka Metropolitan University Graduate School of Medicine Osaka Japan; ^4^ Department of Pediatric Logopedics Osaka City General Hospital Osaka Japan; ^5^ Division of Neurology The Hospital for Sick Children Toronto Ontario Canada

**Keywords:** corpus callosotomy, encephalitis, encephalopathy, late‐onset epileptic spasm, stimulus‐induced/startle seizure, subtotal hemispherotomy

## Abstract

**Objective:**

We aimed to analyze the efficiency of corpus callosotomy (CC) and subsequent disconnection surgeries in patients with late‐onset epileptic spasms (LOES) by comparing post‐encephalitis/encephalopathy (PE) and non‐encephalitis/encephalopathy (NE). We hypothesized these surgeries can control potential focal onset epileptic spasms (ES) in the NE group but not in the PE group.

**Methods:**

We retrospectively included 23 patients (12 with PE and 11 with NE) who initially underwent CC and subsequent disconnection surgeries (five NE). We compared the clinical courses, seizure types, MRI, video‐EEG, epilepsy surgery, and seizure outcomes between the two groups.

**Results:**

The median age of LOES onset in the PE group was 2.8 (range 1.0–10.1 years) and 2.9 years (range 1.1–12.6) in the NE group. Bilateral MRI abnormalities were observed in both groups (PE, n = 12; NE, n = 3; *P* < 0.05). The PE group presented ES alone (n = 2), ES + focal seizures (FS) (n = 3), ES + generalized seizures (GS) (n = 3), and ES + FS + GS (n = 4) in addition to stimulus‐induced startle seizures (SS) (n = 8) (mean 3.1 seizure types/patient). The NE group presented ES alone (n = 1), ES + FS (n = 2), and ES + FS + GS (n = 8) (mean 2.7 seizure types/patient). In the PE group, CC stopped ES (n = 1) and SS (n = 1) and achieved <50% SS (n = 3). In the NE group, CC achieved immediate ES‐free status (n = 2) and < 50% ES (n = 1), and additional disconnection surgeries subsided all seizure types (n = 3) based on lateralized interictal/ictal EEG findings. LOES was significantly remitted by surgery in the NE group (6/11 [55%]) compared with the PE group (1/12 [8%]) (*P* < 0.05).

**Significance:**

LOES is a drug‐resistant, focal/generalized/unknown onset ES. Lateralization of ES in NE could be achieved after CC and eliminated by further disconnection surgeries because of potential focal onset ES. LOES in PE had little benefit from CC for generalized onset ES. However, CC might reduce SS in patients in the PE group with multiple seizure types.

**Key Points**

- Late‐onset epileptic spasms (LOESs) are drug‐resistant, focal/generalized/unknown onset epileptic spasms (ES).
- LOES patients may achieve lateralization of ES after corpus callosotomy (CC) and elimination by further disconnection surgeries.
- CC might reduce stimulus‐induced/startle seizures in the post‐encephalitis/encephalopathy group.

## 1 INTRODUCTION

Late‐onset epileptic spasms (LOESs) are defined as epileptic spasms (ES) that start after the first year of life. The occurrence rate of LOES is estimated to be 2%–8% of ES, including all age groups.^1^, ^2^, ^3^ LOESs are poorly understood because of their heterogeneous etiologies, clinical features, and differing treatments. The effects of antiseizure medications (ASMs) and adrenocorticotrophic hormone (ACTH) therapy for LOES are more limited than those for infantile epileptic spasms, and controlling LOES is often difficult.^4^, ^5^, ^6^

The etiologies of LOES can be divided into two major groups: post‐encephalitis/encephalopathy (PE group) and non‐encephalitis/encephalopathy (NE group). In both the PE and NE groups, electroencephalography (EEG) often shows bilateral hemispheric abnormalities, and the semiology of ES may not be lateralized as in most LOES. Therefore, corpus callosotomy (CC) has been applied after the diagnosis of lateralization and is a part of treatment for LOES in Japan, based on the theory that secondary generalization could occur by the transcallosal response, as observed in experiments in *Papio papio*.^7^, ^8^, ^9^, ^10^

We hypothesized that a subset of patients with LOES in the NE group would benefit from CC to lateralize the ES, followed by disconnection surgeries to suppress their epileptic network. CC may have little effect on stimulus‐induced startle seizures (SS), which are frequently observed in multiple types of drug‐resistant seizures in the PE group. However, CC would not be expected to achieve the same benefit in the PE group as in the NE group. To our knowledge, few studies have focused on epilepsy surgery in LOES^8^, ^9^, ^10^, ^11^ and none have comprehensively examined the etiologies of LOES and epilepsy surgery. This study aimed to assess the various clinical features and seizure outcomes of CC for LOES by comparing the PE and NE groups.

## 2 MATERIALS AND METHODS

### 2.1 Patients

**FIGURE 1:**
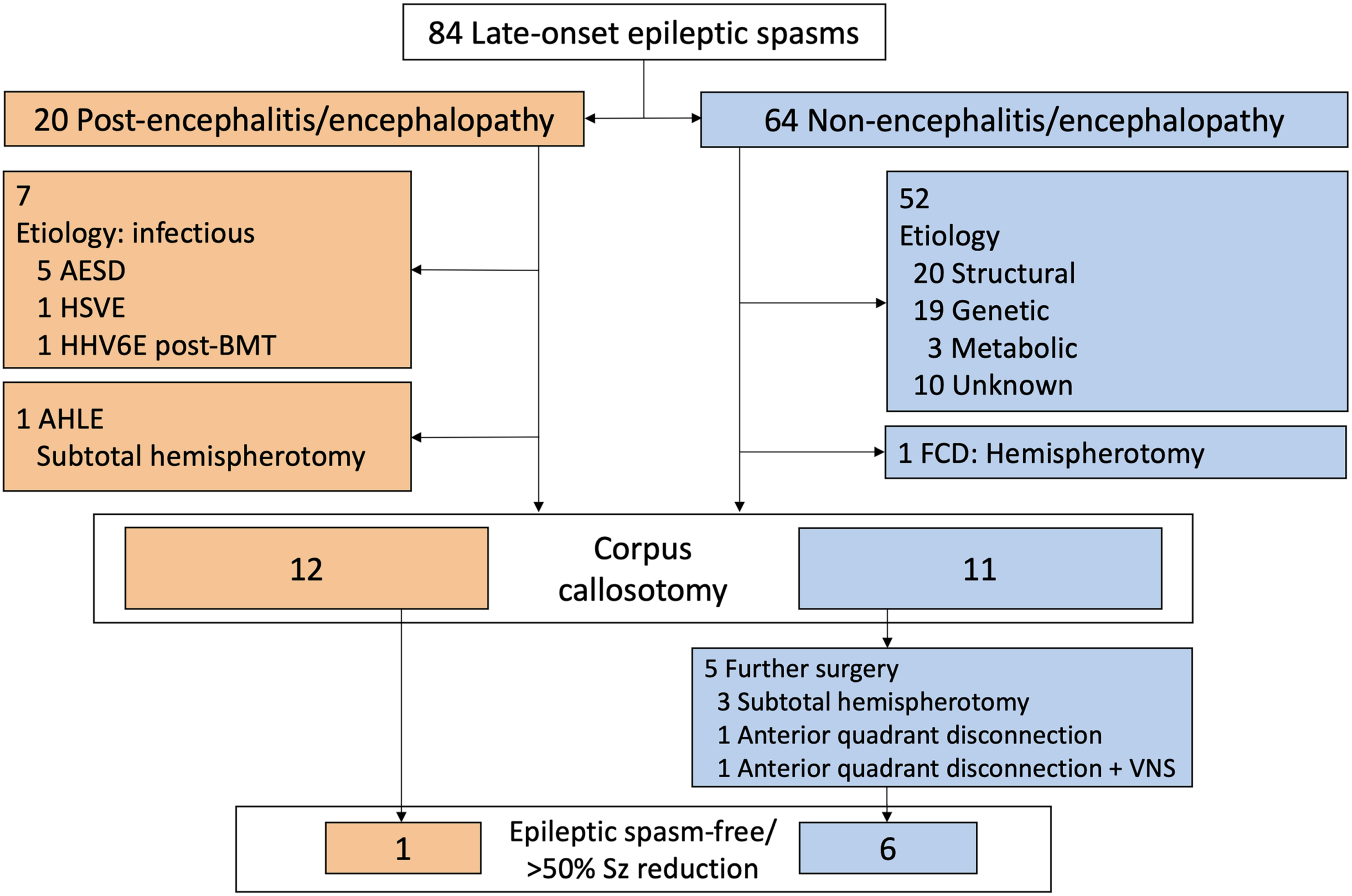
Study flow chart. AESD, acute encephalopathy with biphasic seizures and late reduced diffusion; AHLE, acute hemorrhagic leukoencephalitis; FCD, focal cortical dysplasia; HHV6E post‐BMT, human herpesvirus 6 encephalitis post–bone marrow transplantation; HSVE, herpes simplex virus encephalitis; Sz, seizure; VNS, vagus nerve stimulation

We retrospectively included 23 pediatric patients with LOES who underwent CC with and without subsequent epilepsy surgery at Osaka City General Hospital from June 1995 to December 2021. During this period, we collected a total of 84 patients with LOES (20 and 64 secondary to PE and NE, respectively) (Figure 1). In the 64 patients in the NE group, epilepsy surgery was performed in 12 patients (19%), of which 11 patients underwent initial CC during this study period, and one patient with focal cortical dysplasia (FCD) with hemiplegia underwent one‐stage hemispherotomy with a seizure‐free outcome. The remaining 52 patients did not undergo CC. The etiologies comprised structural abnormalities (perinatal injury, suspected FCD, brain tumor, hydrocephalus, and others) in 20 patients, genetic etiologies in 19 patients, metabolic disorders in 3 patients, and unknown etiologies in 10 patients.

Of the 20 patients in the PE group, CC was performed in 12 patients (60%). Seven patients who did not undergo CC comprised acute encephalopathy with biphasic seizures and late reduced diffusion (AESD) in five patients; herpes simplex virus encephalitis in one patient, and human herpesvirus 6 encephalitis post–bone marrow transplantation (HHV6E post‐BMT) in one patient. One patient underwent subtotal hemispherotomy for acute hemorrhagic leukoencephalitis; however, the seizures persisted.

The 23 pediatric patients with LOES who underwent CC with and without subsequent epilepsy surgery were divided into two groups:12 patients in the PE group and 11 in the NE group. In the PE group, we excluded patients with known genetic disorders, congenital brain malformations, metabolic disorders, perinatal encephalopathy, and other neurological disorders diagnosed prior to acute onset, infection‐related encephalitis/encephalopathy. Patients were investigated for etiologies, clinical characteristics, seizure types, response to ASMs, ketogenic diet, ACTH therapy, timing and procedures of the epilepsy surgery, outcomes, and sequelae. The investigations included magnetic resonance imaging (MRI) and video‐EEG. ACTH therapy consisted of injection of synthetic ACTH (a zinc hydroxide suspension of tetracosactide acetate [Cortrosyn‐Z]) at a dose of 0.0125 mg/kg every day for 2 weeks (maximum, 3 weeks), followed by tapering for 1 or 2 weeks.

We performed preoperative and postoperative assessments of intellectual disability and motor dysfunction. Intellectual disability was classified based on the intelligence quotient as most severe [–19], severe [20–34], moderate [35–49], mild [50–69], and borderline [70–79].

### 2.2 Seizures

Scalp electrodes were placed according to the international 10–20 system. Surface electromyography electrodes were placed bilaterally on the deltoid muscles. Video‐EEG was recorded with a bandpass filter of 0.08–60 Hz and a sampling rate of 200 Hz (EEG 9100 and 1200, Nihon Kohden). Electrocorticography (ECoG) was recorded with a bandpass filter of 0.016–600 Hz and a sampling rate of 2000 Hz (EEG 1200, Nihon Kohden).

Epileptic spasms in all patients was confirmed by video‐EEG, showing diffuse high‐voltage slow waves with fast activity manifesting as brief contractions of axial and proximal muscles of the extremities, resulting in muscle flexion, extension, or a mixture of both.

Startle seizures are defined as focal/generalized/unknown onset tonic seizures precipitated by sudden and unexpected auditory, tactile, and other stimuli.^12^, ^13^ In terms of frequent traumatic falls and severe decline in the quality of life, SS were evaluated separately from other seizures in this study.

### 2.3 Surgeries

At our institution, the surgical strategy was discussed for each patient by our institutional epilepsy board. CC is considered the first step in epilepsy surgery in patients with ES who show bilateral synchronous EEG abnormalities and are drug‐resistant to ASMs and ACTH therapy. Total CC (TCC) was chosen for patients under 10 years of age, and for those over 10 years of age, TCC or anterior 2/3 CC (ACC) was selected to reduce disconnection syndromes, considering cognitive impairment in each patient.^14^, ^15^ If lateralization of the EEG is confirmed after CC, the subsequent epilepsy surgery, that is, subtotal hemispherotomy, anterior quadrant disconnection (AQD), or posterior quadrant disconnection (PQD), is actively considered to eliminate all types of seizures. Subtotal hemispherotomy consists of AQD, PQD, and CC of the central part after undergoing intracranial video‐EEG using chronic subdural grid electrodes to identify the seizure network and confirm the primary motor area (PMA) could be preserved.^16^, ^17^, ^18^ In a subset of patients with apparent unilateral MRI lesions and EEG abnormalities, subtotal hemispherotomy, hemispherotomy, or other disconnection surgeries would be chosen, considering age and presence, degree, and extent of hemiplegia.

### 2.4 Evaluation of surgical outcomes

Outcomes after CC and subsequent surgery were evaluated with reference to the International League Against Epilepsy classification of postoperative seizure outcome^19^: seizure‐free: (class 1); seizure >50% reduction in the baseline number of seizures: classes 2–4; seizure <50% reduction in the baseline number of seizures: classes 5 and 6. The reduction rates for ES and all seizure types were calculated by dividing the postoperative ES and all seizure types by the preoperative ES and all seizure types in each group.

### 2.5 Statistical analysis

Continuous data are presented as median and range. Categorical data are presented as numbers. Between‐group comparisons for continuous variables were made using the Mann–Whitney U test and for categorical variables using the Fisher's exact test. Differences were considered statistically significant at *P* < 0.05. All statistical analyses were performed using SPSS version 22.0 for Windows (IBM Japan).

### 2.6 Ethical approvals

This study was approved by the ethics committee of Osaka City General Hospital (Nos. 1607034 and 1611075). Written informed consent for this study and its publication was obtained from the patients' parents.

## 3 RESULTS

### 3.1 Patients and clinical characteristics

Tables 1 and 2 summarize the clinical characteristics, medical treatments, MRI findings, seizure types, and epilepsy surgery, comparing the PE and NE groups.

**TABLE 1:** Clinical characteristics, MRI, seizure types, epilepsy surgeries

	PE (n = 12)	NE (n = 11)	*P*‐value
Male/Female	9/3	5/6	0.214
Age at acute encephalitis/encephalopathy onset (y)	1.4 (0.5–9.9)	–	
Age at late‐onset epileptic spasms (y)	2.8 (1.0–10.1)	2.9 (1.1–12.6)	0.926
Mean ASMs in history (range)	11 (3–24)	11 (4–16)	0.757
Adrenocorticotrophic hormone therapy	5	8	0.214
Intellectual disabilities (pre‐CC)			0.003
Most severe	11	3	
Severe	0	3	
Moderate	1	2	
Mild	0	1	
Border	0	2	
Motor deficits tetraplegia	7	1	0.027
MRI findings			
Abnormalities	12	5	0.005
Bilateral/unilateral	12/0	3/2	0.074
Interictal EEG			
Multifocal spikes	10	10	>0.999
Generalized spikes	8	6	0.680
Seizures			
ES	2	1	>0.999
ES + FS	3	2	>0.999
ES + GS	3	0	0.217
ES + FS + GS	4	8	0.100
SS	8	0	0.001
Epilepsy surgeries			
CC	12	11	–
CC + Subtotal hemispherotomy	0	3	0.093
CC + Anterior quadrant disconnection	0	1	0.478
CC + Anterior quadrant disconnection + VNS	0	1	0.478
Mean follow‐up period (y)	10.9 (3.6–26.9)	11.1 (3.2–24.6)	0.712

Abbreviations: ASMs, antiseizure medications; CC, corpus callosotomy; ES, epileptic spasms; FS, focal seizures; GS, generalized seizures; NE, non‐encephalitis/encephalopathy; PE, post‐encephalitis/encephalopathy; SS, stimulus‐induced startle seizure; VNS, vagus nerve stimulation.

**TABLE 2:** Epilepsy surgeries and last seizure outcome

No. of patients	Etiology	Age of LOES (y)	Pre‐ID	Latest ID	Tetraplegia	MRI		Interictal EEG		Corpus callosotomy	Age of CC (y)	Additional surgeries	Age of additional surgeries	Last follow‐up Sz				Follow‐up (y)
						Lesion	Bilateral/unilateral	Multifocal	Generalized					ES (11)	FS (6)	GS (7)	SS (4)	
PE																		
1	HSES	5.4	MS	MS	+	+	Bilateral	FP	+	Total CC	9.1	None	−	Sz free	−	−	−	9.9
2	HHV6E post‐BMT	9.5	M	M	−	+	Bilateral	FPT	+	Total CC	16.3	None	−	NO	NO	NO	Sz free	11.9
3	FIRES	2.6	MS	MS	+	+	Bilateral	FT	−	Total CC	5.9	None	−	NO	NO	NO	>50%	12.0
4	AESD‐CS	4.4	MS	MS	+	+	Bilateral	FPT	−	Total CC	7.6	None	−	NO	NO	NO	>50%	17.0
5	AESD‐D	1.6	MS	MS	−	+	Bilateral	FCPTO	+	Total CC	5.2	None	−	NO	−	NO	>50%	5.9
6	AESD‐CS	5.0	MS	MS	+	+	Bilateral	FPT	+	Anterior CC	22.9	None	−	NO	Sz free	NO	NO	22.3
7	AESD‐D	1.5	MS	MS	−	+	Bilateral	−	+	Total CC	2.7	None	−	NO	Sz free	NO	NO	6.1
8	AESD‐CS	1.2	MS	MS	−	+	Bilateral	FPT	−	Total CC	9.5	None	−	NO	−	Sz free	NO	8.7
9	Herpes simplex virus encephalitis	1.0	MS	MS	−	+	Bilateral	FTO	+	Total CC	6.7	None	−	NO	NO	Sz free	−	13.1
10	AESD‐D	10.1	MS	MS	+	+	Bilateral	FCPO	+	Anterior CC	13.6	None	−	NO	NO	NO	NO	5.2
11	AESD‐CS	2.3	MS	MS	+	+	Bilateral	FPO	−	Total CC	3.0	None	−	NO	−	−	−	3.6
12	AESD‐CS	3.0	MS	MS	+	+	Bilateral	−	+	Anterior CC	25.1	None	−	NO	NO	−	−	26.9
													>50% Sz reduction rate	8%	25%	22%	50%	
NE														**ES (5)**	**FS (8)**	**GS (5)**	**SS (0)**	
1	FCD	1.4	S	M	−	−	−	FPT	−	Total CC	2.6	Subtotal hemispherotomy	4.3	Sz free	−	−	−	5.5
2	FCD	6.2	Mi	Mi	−	−	−	FPT	−	Total CC	12.4	Subtotal hemispherotomy	15.7	Sz free	Sz free	Sz free	−	11.1
3	FCD	2.9	B	B	−	−	−	FPT	+	Total CC	13.8	Subtotal hemispherotomy	15.3	Sz free	Sz free	Sz free	−	15.3
4	FCD	12.6	B	B	−	+	Unilateral	FP	−	Anterior CC	13.9	AQD	15.3	Sz free	NO	Sz free	−	3.5
5	FCD	1.5	M	M	−	−	−	FP	+	Total CC	4.8	AQD, VNS	6.7, 7.8	>50%	NO	−	−	13.0
6	Subcortical band heterotopia	3.9	M	Mi	−	+	Bilateral	FCPTO	−	Total CC	4.7	None	−	Sz free	NO	NO	−	7.8
7	Subcortical band heterotopia	12.1	S	S	−	+	Bilateral	FCPT	+	Anterior CC	14.1	None	−	NO	NO	NO	−	5.8
8	Neurocutaneous disorder	1.2	S	S	−	+	Unilateral	FPT	+	Total CC	2.9	None	−	NO	NO	−	−	3.2
9	Perisylvian syndrome	10.5	MS	MS	+	+	Bilateral	−	+	Total CC	16.7	None	−	NO	NO	NO	−	12.1
10	Unknown	1.2	MS	MS	−	−	−	FPT	−	Total CC	8.9	None	−	NO	NO	NO	−	20.7
11	Unknown	1.1	MS	MS	−	−	−	FPT	+	Total CC	6.7	None	−	NO	NO	NO	−	24.6
													>50% Sz reduction rate	55%	20%	38%	−	

Abbreviations: AESD‐CS, acute encephalopathy with biphasic seizures and late reduced diffusion‐central sparing pattern; AESD‐D, AESD‐diffuse pattern; AQD, anterior quadrant disconnection; ASMs, antiseizure medications; CC, corpus callosotomy; EEG, electroencephalography (F—frontal, C—central; P—parietal; T—temporal; O—occipital); ES, epileptic spasms; FCD, focal cortical dysplasia; FIRES, febrile infection‐related epilepsy syndrome; FS, focal seizures; GS, generalized seizures; HHV6E post‐BMT, human herpesvirus 6 encephalitis post–bone marrow transplantation; HSES, hemorrhagic shock and encephalopathy syndrome; ID, intellectual disability (IQ: MS—most severe [−19]; S—severe [20–34]; M—moderate [35–49]; Mi—mild [50–69]; B—border [70–79]); LOES—late‐onset epileptic spasm; NE, non‐encephalitis/encephalopathy; NO, no change; PE, post‐encephalitis/encephalopathy; SS, stimulus‐induced startle seizure; Sz, seizure; VNS, vagus nerve stimulation.

The median age of LOES in the PE group was 2.8 (range 1.0–10.1 years), whereas it was 2.9 years (range 1.1–12.6) in the NE group. In both groups, ASMs (including vigabatrin for two patients in the PE group and three in the NE group) and a ketogenic diet (two patients in the PE group and one in the NE group) did not lead to cessation of seizures. Thirteen patients received ACTH therapy (five in the PE group and eight in the NE group). Twelve of the 13 patients relapsed with ES within a year. One patient experienced ES after a four‐year seizure‐free interval.

In the PE group, eight patients had AESD. The other four patients had herpes simplex virus encephalitis, hemorrhagic shock and encephalopathy syndrome (HSES), febrile infection‐related epilepsy syndrome, and HHV6E post‐BMT. In the NE group, five patients had FCD, two had subcortical band heterotopia (SBH), one each had a neurocutaneous disorder and perisylvian syndrome, and the other two had unknown etiologies. All patients in both groups had borderline‐to‐most severe cognitive impairment. The number of patients with the most severe cognitive impairment and tetraplegia were significantly higher in the PE group than in the NE group.

### 3.2 MRI findings

**FIGURE 2:**
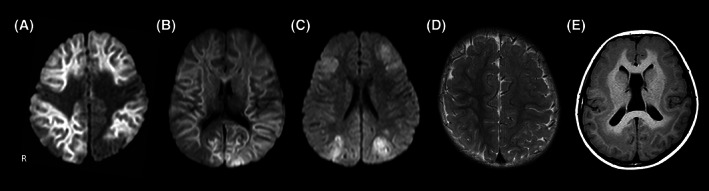
Brain MRI findings, A, AESD: Diffusion‐weighted imaging (DWI) image shows high signal intensity in the bilateral diffuse subcortical white matter, with relatively central sparing in the acute phase. B, AESD: DWI image shows bilateral diffuse subcortical high signal intensity without central sparing in the acute phase. C, HSES: DWI image shows symmetrical abnormally high signal intensity in the cortex and subcortical white matter in the acute phase. D, FCD: T2‐weighted image shows left hemispheric volume loss, abnormal cortical thickening, and blurring of the gray‐white matter junction. E, SBH: T1‐weighted image shows bilateral subcortical heterotopic gray matter in the posterior region fused with the pachygyric cortex in the anterior region. AESD, acute encephalopathy with biphasic seizures and late reduced diffusion; DWI, diffusion‐weighted imaging; FCD, focal cortical dysplasia; HSES, hemorrhagic shock and encephalopathy syndrome; MRI, magnetic resonance imaging; R, right; SBH, subcortical band heterotopia

All patients in the PE group demonstrated bilateral MRI abnormalities during the acute phase (Figure 2A–C). Five patients with AESD showed diffuse involvement, relatively sparing the PMA (Figure 2A); the other three patients with AESD showed diffuse subcortical white matter involvement (Figure 2B).

In the NE group, five patients showed MRI abnormalities (bilateral, 3; unilateral, 2). In one patient with a unilateral MRI abnormality, MRI findings of abnormal cortical structure, cortical thickening, and blurring of the gray‐white matter junction suggested FCD (Figure 2D). The other patient with a unilateral MRI abnormality had a neurocutaneous disorder. Bilateral MRI abnormalities were found in two patients with SBH (Figure 2E) and one with perisylvian syndrome.

### 3.3 EEG findings

**FIGURE 3:**
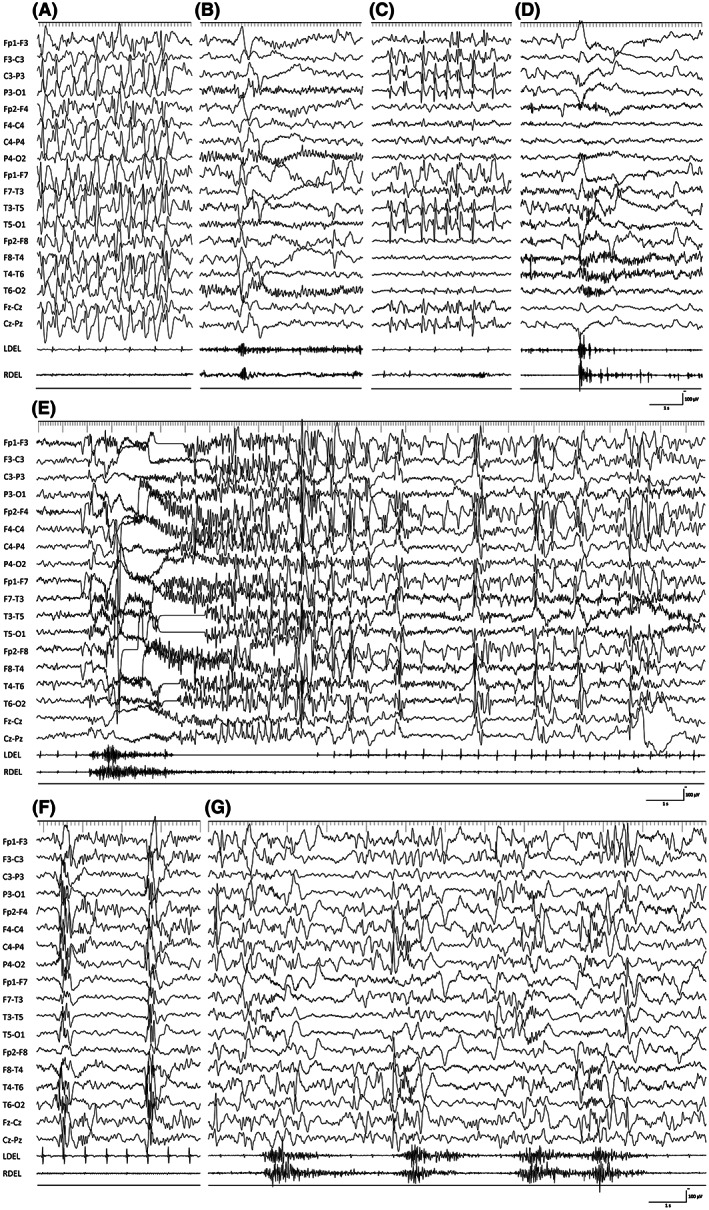
EEG of representative patients. A–D, EEG of a representative patient who underwent TCC, followed by subtotal hemispherotomy, led to seizure‐free. A, Interictal EEG before TCC shows bilateral multifocal spike‐and‐wave complexes. B, Ictal EEG before TCC shows diffuse high‐voltage slow waves superimposed with fast waves without preceding polyspike‐and‐wave complexes. C, Interictal EEG post‐TCC shows lateralized spike‐and‐wave complexes in the left hemisphere. D, Ictal EEG post‐TCC shows diffuse high‐voltage slow waves superimposed with fast waves, dominant in the left hemisphere. E, EEG of a representative patient with SS before TCC. Ictal EEG before TCC shows high‐voltage bilateral frontal‐dominant polyspike‐and‐wave complexes followed by sustained (>2 s) slow waves with low‐voltage fast waves. This finding remains unchanged even after TCC. F and G, EEG of a representative patient with persistent ES after TCC. F, Interictal EEG after TCC shows persistent bilateral synchronous polyspike‐and‐wave complexes. G, Ictal EEG after TCC shows bilateral diffuse high‐voltage slow superimposed with fast waves. Even within an identical series of ES, left‐ and right‐side dominant ES are observed. CC, corpus callosotomy; EEG, electroencephalography; ES, epileptic spasms; TCC, total CC

Interictal epileptic discharges were multifocal spike‐and‐wave complexes in 10 patients each, both in the PE and NE groups (Tables 1 and 2 and Figure 3A). Generalized spike‐and‐wave complexes were observed in eight patients in the PE group and six in the NE group. None of the patients showed clear laterality and hypsarrhythmia.

Ictal EEG of LOES before CC showed diffuse high‐voltage slow waves superimposed with fast waves in all patients, with no preceding polyspike‐and‐wave complexes (Figure 3B). After TCC, three patients in the NE group showed lateralized interictal and ictal EEG; therefore, they underwent subtotal hemispherotomy (Figure 3C,D). Ictal EEG of SS was characterized by high‐voltage bilateral frontal‐dominant polyspike‐and‐wave complexes followed by sustained (>2 s) slow waves with low‐voltage fast waves (Figure 3E). Even after CC, 11 patients in the PE group and 5 in the NE group had persistent bilateral synchronous/asynchronous independent EEG abnormalities observed prior to CC (Figure 3F,G).

### 3.4 Seizure types

The PE group presented with ES alone (n = 2), ES+ focal seizures (FS) (n = 3), ES + generalized seizures (GS) (n = 3), ES + FS + GS (n = 4), and SS (n = 8) (mean 3.1 seizure types/patient). SS were activated only by sound (n = 5), sound+touch (n = 2), or sound+visual stimuli (n = 1). The NE group presented ES alone (n = 1), ES + FS (n = 2), ES + FS + GS (n = 8) (mean 2.7 seizure types/patient). All patients in both groups presented with symmetrical ES without consistent laterality signs on clinical semiology and electromyography of the deltoid muscles.

Startle seizures in eight patients in the PE group presented characteristically focal/generalized/unknown onset seizures with or without awareness that lasted <30 seconds, with an initial startle response followed by axial bilateral tonic posturing.

### 3.5 Epilepsy surgery and seizure outcomes

We performed CC between 2.6 and 25.1 years with a mean age of 8.9 years (Table 2). Eighteen patients underwent TCC (nine in the PE group and nine in the NE group). The remaining five patients underwent ACC (three in the PE group and two in the NE group). The median age of TCC was 6.7 years ranging from 2.6 to 16.7 years. The median age of the ACC was 14.1 years ranging from 13.6 to 25.1 years.

In the 11 patients in the NE group, ES disappeared in two patients consisting of one with FCD after ACC and the other with SBH after TCC, while FS persisted. In the remaining nine patients, the frequency of ES was not reduced to ≤50%. Additional disconnection surgeries were performed in five patients; three patients with subtotal hemispherotomy after TCC suppressed all seizure types, and interictal/ictal EEG findings were lateralized. The other two patients underwent AQD. One patient became ES‐free but had persistent FS after ACC. The other patient underwent vagus nerve stimulation after TCC for the remaining ES and FS without a successful outcome. LOES was significantly controlled by surgery in 6/11 (55%) patients in the NE group compared with the PE group, 1/12 (8%) (*P* < 0.05). The >50% seizure reduction rates for all seizure types (66 types: 29 types in the NE group and 37 types in the PE group) included 37.9% in the NE group and 24.3% in the PE group.

### 3.6 Comorbidity

The postoperative courses were uneventful, and none of the patients had definite disconnection syndrome. Two of the three patients who underwent subtotal hemispherotomy after TCC presented with clumsy hand movements. No hemiplegia was observed in the three patients who underwent subtotal hemispherotomy.

### 3.7 Pathology

Four patients had FCD without MRI abnormalities, confirmed as FCD type 1 in the pathological analysis after epilepsy surgery.

## 4 DISCUSSION

Summary of findings: (1) In the NE group (11 patients), CC immediately suppressed ES in two and reduced to <50% in one patient, and three patients became seizure‐free following additional disconnection surgeries after CC, who remained ES >50% after CC but had lateralized EEG abnormalities; (2) In the PE group (12 patients), CC caused patients to be ES‐free (n = 1), SS‐free (n = 1), and SS <50% (n = 3). Even after CC, 11 of the 12 PE patients had persistent bilateral EEG abnormalities and the same seizure types, which prevented to proceed to the next epilepsy surgery.

### 4.1 Hidden potential focal onset LOES in the NE group

A subset of patients in the NE group achieved lateralization of EEG abnormalities and LOES after CC, and further disconnection surgeries may eliminate potential focal onset ES.

The etiology of LOES in the NE group was heterogeneous. The structural etiology of the NE group with LOES mainly displayed FCD.^6^, ^11^, ^20^, ^21^ Genetic disorders include *MECP2* gene duplication^22^ and *CASK* gene mutation.^6^, ^23^ Metabolic syndrome has also been reported.^6^, ^24^

Metsahonkala et al.^11^ performed epilepsy surgery in six (five with FCD; one with unknown) out of 17 patients with LOES. Five sublobar resections and one multilobar resection led to seizure‐free status in four patients and persistent FS in two. Baba et al. reported that 56 patients with drug‐resistant ES, including those with both infantile epileptic spasms and LOES, underwent CC. EEG after CC showed lateralized or localized EEG abnormalities in 19 patients (33.9%).^8^ Twelve of the 19 patients underwent subsequent surgery consisting of functional/subtotal hemispherotomy (n = 3), AQD (n = 4), and PQD (n = 5), resulting in favorable outcomes.^8^, ^9^ Recently, Uchida et al.^25^ have delineated that neck flexion and muscular contraction of the extremities in ES had more asymmetric expression after CC. The post‐CC asymmetrical semiology could be an important lateralizing sign indicating the need for further disconnection surgery to control ES. However, before CC, <40% of patients showed asymmetrical ES and 30% showed unilaterally predominant EEG discharges.

**FIGURE 4:**
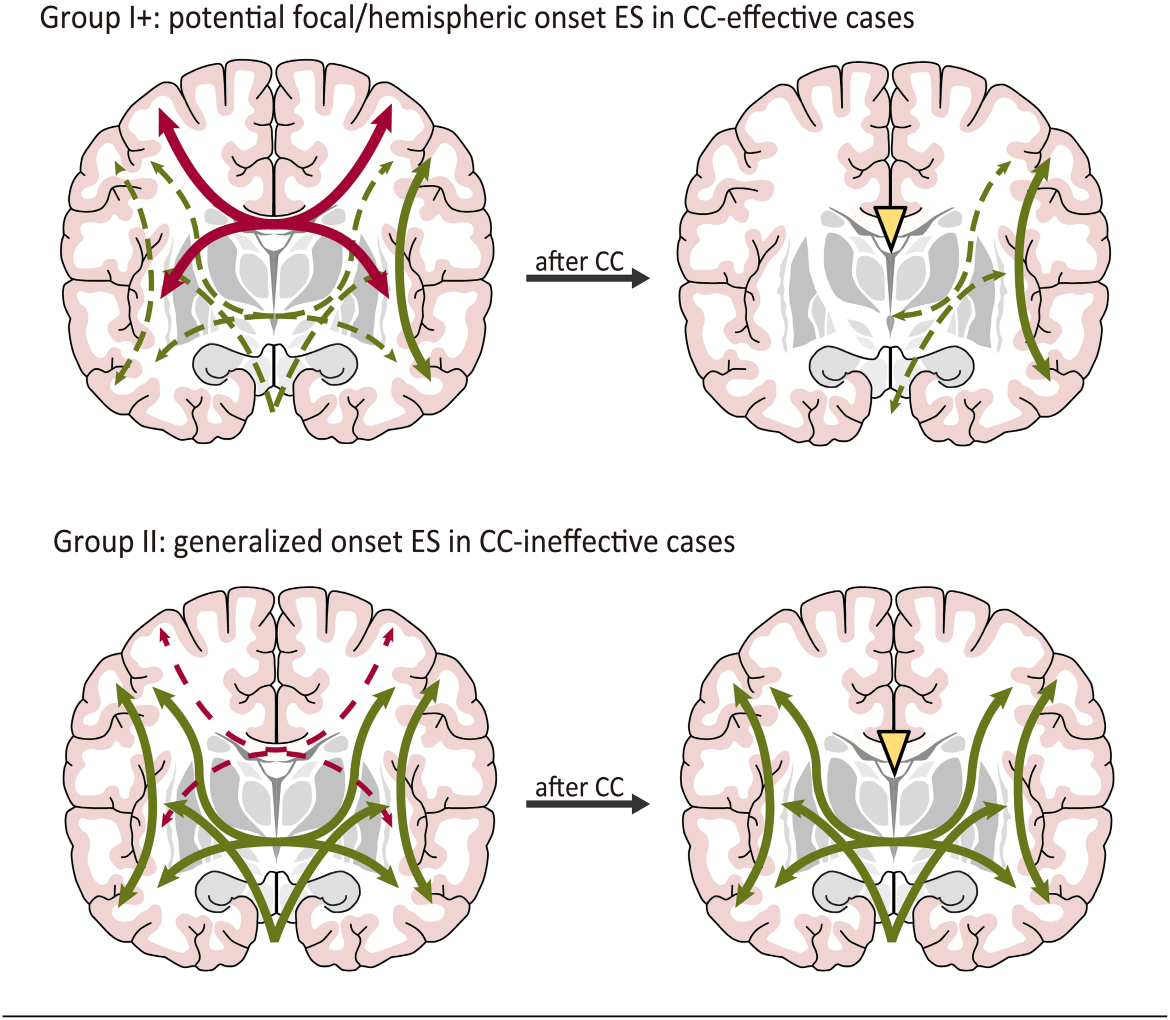
Schema of potential mechanisms underlying the generation of ES. Group I+: potential focal/hemispheric onset ES in CC‐effective cases. High interhemispheric modulation of the corpus callosum (thick red lines) leads to rapid bilateral propagation of epileptic excitations, provoking symmetrical ES. The brainstem and subcortical regions (dashed green lines) may play smaller roles than the corpus callosum. After CC (yellow triangle), this group has a chance to achieve lateralization of EEG abnormalities and ES, and further surgeries to disconnect the intrahemispheric epilepsy network (green line) can eliminate ES. Group II: generalized onset ES in CC‐ineffective cases. The callosal modulation (dashed red lines) may be lower, and the subcortical involvement (thick green lines) may be higher than in CC‐effective cases. Even after CC (yellow triangle), patients in this group could not proceed to the subsequent epilepsy surgery

Three groups of ES can be defined based on intra‐ and interhemispheric modulation, as proposed by Uda et al.^26^ Group I is defined as focal onset ES observed from the first diagnosis and showing focal or lateralized EEG abnormalities with or without MRI lesions. These patients require focal disconnection without CC. No group I ES was included in this study. Group I, which displays high interhemispheric modulation of the corpus callosum (Group I+; potential focal onset ES), required CC and subsequent disconnection surgery in our study (Figure 4, group I +). The three patients in the NE group who were seizure‐free after CC and additional disconnection surgeries belonged to Group I+. Patients fulfilling the concept of potential focal onset ES proposed by Okanishi and Fujimoto^27^ were also considered to belong to this group. Eleven patients in the PE group and four in the NE group belonged to Group II (generalized onset ES). In this group, ES and multiple seizures are under the extensive epilepsy network of intra‐/interhemispheric modulations and possible brainstem and subcortical regions^28^, ^29^, ^30^ (Figure 4, Group II). This group could not proceed to the subsequent epilepsy surgery, even after CC. The immediate ES‐free state after CC in the NE group and one patient in the PE group could be classified as Group III, in which predominant interhemispheric modulation of the corpus callosum provokes LOES. The corpus callosum of the latter patient, who presented only LOES after HSES, might have a significantly higher modulation role that provoked ES, indicating lesser cortical/subcortical roles for ES, even post‐HSES.

The corpus callosum is considered to be highly modulated to provoke ES from one hemisphere to the other and represents a possible subcortical pathway. The efficacy of CC and subsequent disconnection surgeries may depend on the etiology of LOES. The corpus callosum may play a different role in the transcallosal network during LOES in the NE and PE groups.

In a subset of the NE group, PMA was spared because of early myelination before birth, whereas the association fiber pathways become gradually myelinated after birth.^31^, ^32^, ^33^ Recent integrated neurophysiological techniques have provided converging evidence for the involvement of abnormal cortico‐cortical networks in multilobar epileptic zones, relatively sparing the PMA in the generation of ES.^16^, ^17^, ^33^, ^34^, ^35^, ^36^ Subtotal hemispherotomy could be considered the last resort for patients with drug‐resistant LOES in the NE group.

### 4.2 Patients in the PE group experience fewer benefits from CC than the NE group for generalized onset ES

The etiologies of LOES in the PE group consisted of eight patients with AESD,^6^, ^37^ two with other viral encephalitis,^22^ and one each with HSES^38^ and febrile infection‐related epilepsy syndrome.^39^ Ito et al.^37^ reported that of 44 patients who underwent AESD, 10 (23%) had post‐encephalopathic epilepsy. Four of the 10 patients presented with LOES drug‐resistant to ASMs.

In the NE group with LOES, CC could be considered for potential focal onset ES (Group I+); however, for LOES in the PE group, CC had fewer benefits than for the NE group. The main etiology of our PE group presenting with LOES was infection/encephalitis and structural/bilateral MRI abnormalities. The bilateral hemispheres, including cortical and subcortical structural abnormalities, provoked generalized onset ES (Group II). CC did not suppress the LOES in 83% of the patients in the PE group. The interhemispheric corpus callosum of patients in the PE group may play a smaller role in modulation than bilateral hemispheric abnormalities, such as intrahemispheric and subcortical regions, to provoke LOES (Figure 4, Group II).

In patients in which CC was ineffective, in most of the PE group and a subset of the NE group, callosal modulation may be lower and subcortical involvements may be higher than in CC‐effective cases.

### 4.3 CC to eliminate or reduce SS in PE group

It was worth considering CC for reducing SS in the PE group, which was often drug‐resistant to medical treatments and seriously impaired daily life.

Most patients with SS usually have multilobar lesions secondary to pre‐ or perinatal insults or structural/infection etiologies within the first 2 years of life.^13^ They suffered a severe head injury due to sudden tonic posturing and head drops, which reduced their quality of life. In the PE group, eight (67%) of the 12 patients presented with SS, in addition to FS (n = 6) and GS (n = 7). SS tended to occur in patients with multiple types of drug‐resistant epilepsy, as in our cases.

Combined magnetoencephalography and video‐EEG study delineated the SMA and cingulate gyrus as regions that generate SS.^40^, ^41^ The seizure semiology of SS observed in this series was similar to tonic sudden bilateral SMA seizures. EEG abnormalities were diffuse in SS cases in the PE group. SMA onset SS could not be proven in our cases unless we performed ECoG to include the SMA.

For the PE group, LOES and SS were drug‐resistant, even ACTH, which is the last resort for ES in infantile epileptic spasms. There were a few cases of SS controlled by CC in this series. One patient achieved complete remission of LOES, and three patients had <50% SS after CC with improved quality of life, instead of no change in other seizure types. The SMA and corpus callosum might play a major role in provoking SS among bilateral/generalized epileptic networks secondary to encephalitis/encephalopathy. SS could have been eliminated or halved by disengaging the network through the corpus callosum between the bilateral SMAs by CC. Patients with SS have been reported to benefit from surgical interventions, including lesionectomy, CC, or multiple subpial transection.^12^, ^42^, ^43^ However, half of the SS patients failed to achieve a seizure reduction of ≥50%. The seizure activity was probably not traversing through networks between bilateral SMAs through the corpus callosum but through other descending pathways.

In future cases of drug‐resistant SS, a detailed ECoG/intracerebral EEG should be applied to investigate correlations between the SMA and corpus callosum during SS and the effect of disconnection of the corpus callosum. Consequently, CC with partial SMA resection can be used to eliminate SS.

### 4.4 Limitations

Our study had two limitations. First, the sample size is small. Our hospital is a tertiary care center and widely accepts patients with severe acute encephalitis and encephalopathy. Critical care has changed over time, which may have affected the course of epilepsy and EEG. Second, our hospital has been actively promoting epilepsy surgery, especially since 2015. Patients who developed LOES before then tended to have a longer duration between LOES onset and epilepsy surgery, which may have affected their seizure outcomes. We hope to recruit more LOES patients in the PE and NE groups with epilepsy surgery, including CC and disconnection surgeries, for further analysis.

For patients in this study, magnetoencephalography and bilateral intracranial ECoG, single‐photon emission computed tomography, and positron emission tomography have not been completely applied to detect further lateralization (in addition to video‐EEG and MRI). Other currently available investigations might find Group I+ with potential focal onset LOES.

## 5 CONCLUSION

The present study demonstrated that LOES could be drug‐resistant focal/generalized/unknown onset ES. A subset of patients in the NE group achieved lateralization of ES after CC for further disconnection surgeries to eliminate potential focal onset ES. Patients in the PE group had fewer benefits of CC than the NE group for generalized onset ES; however, SS might decline after CC in these patients. The pathogenesis of LOES may involve diverse mechanisms depending on its etiology; therefore, further studies are needed to elucidate the epileptic network.

## AUTHORS' CONTRIBUTIONS

All authors contributed substantially to the submitted manuscript. Takeshi Inoue and Hiroshi Otsubo involved in conception, data collection, manuscript drafting, and revision. Ichiro Kuki, Takehiro Uda, and Noritsugu Kunihiro involved in data collection, technical advice, manuscript drafting, and revision. Ryoko Umaba, Saya Koh, and Megumi Nukui involved in data acquisition. Shin Okazaki involved in study supervision, manuscript drafts, and revisions.

## CONFLICT OF INTEREST STATEMENT

None of the authors has any conflict of interest to disclose.

## ETHICAL APPROVAL

We confirm that we have read the journal's position on issues involved in ethical publication and affirm that this report is consistent with those guidelines. This study was approved by the ethics committee of Osaka City General Hospital (Nos. 1607034 and 1611075).

## PATIENT CONSENT STATEMENT

Written informed consent for this study and its publication was obtained from the patients' parents.

## Data Availability

The data can be shared upon reasonable request to the corresponding author.
